# Effects of social isolation on sleep during the COVID-19 pandemic

**DOI:** 10.5935/1984-0063.20200097

**Published:** 2021

**Authors:** Franklin Escobar-Córdoba, Jairo Ramírez-Ortiz, Jeisson Fontecha-Hernández

**Affiliations:** 1 Universidad Nacional de Colombia, Faculty of Medicine, Psychiatry Department - Bogotá - Bogotá D.C. - Colombia.; 2 Hospital Universitario Nacional de Colombia, Psychiatry Department -Bogotá - Bogotá D.C. - Colombia.; 3 Fundación Sueño Vigilia Colombiana, Management Office - Bogotá - Bogotá D.C. - Colombia.; 4 NYU Long Island School of Medicine, Department of Psychiatry - New York - New York - United States.

**Keywords:** Pandemics, Social Isolation, Coronavirus Infections, Sleep Initiation and Maintenance Disorders, Sleep Wake Disorders, Sleep Hygiene

## Abstract

The current COVID-19 pandemic is a public health emergency that has seriously affected mental health in the general population. Both, studies on previous epidemics and those conducted during the current pandemic have reported a wide range of psychosocial consequences and multiple psychological symptoms as a result of said outbreaks, and among these problems, sleep/wake cycle alterations stand out. Publications addressing this phenomenon have consistently reported that nearly a third of people who experience social isolation develop insomnia, which, in turn, is an important predictor for mental disorders that affect people’s functionality, including anxiety disorders, depression and post-traumatic stress disorder. This reflection paper aims to describe the effects that social isolation may have on sleep in the context of the current COVID-19 pandemic.

## INTRODUCTION

The sleep-wake cycle is subject to multiple factors, mainly exposure to daylight and nighttime darkness, for the latter increases the levels of melatonin, a hormone that plays a key role in sleep onset and its regulation^1^. Other factors involved in the sleep-wake cycle include mealtimes and daytime physical activity; somehow, due to the current pandemic, daytime physical activity has been affected in two possible ways: people may have, on the one hand, lower physical activity levels due to the mandatory confinement established in most countries, and the depression resulting from this situation; on the other, high levels of activity due to stress, overwork or performing high-intensity exercises at night; in both cases, sleep patterns are affected^1^.

In addition, stress implies a greater psychological and physiological activation in response to the increasing daily activities and responsibilities, and it is well known that the increased function of the hypothalamic-pituitary-adrenal (HPA) axis is associated with shortened sleep and sleep fragmentation, with a possible reduction of the N3 sleep stage^2^. In turn, shortened sleep leads to higher levels of stress-related markers such as cortisol, and, therefore, the resulting sleep disorders may lead to an increased activation of the HPA axis, exacerbating the effects of stress, thus creating a stress-insomnia vicious cycle^2,3^. This bidirectional relationship has been confirmed in studies conducted in workers exposed to chronic occupational stress and who have a higher incidence of sleep disorders, and in those who sleep little or have poor sleep quality, where it has been reported they show more stress, exhaustion, and depression related symptoms than those who sleep well^4^.

Among these studies, it is worth noting the experimental results by Garbarino and Magnavita (2019)^4^, which suggest that sleep is a mediating factor in the relationship between stress and cardiovascular risk, thus concluding that shorter sleep duration and poor sleep quality are useful predictors of biological indicators alterations such as altered levels of blood lipids and heart rate variations; specifically, these results support the hypothesis that sleep problems “having trouble to fall asleep, experiencing interrupted sleep, and early awakenings” are associated with metabolic syndrome^4^.

The sleep plays a fundamental role in the regulation of emotions, since an alteration in its pattern may have direct consequences on the emotional functioning of any individual the day after said alteration occurs^5^; for example, insomnia can increase anxiety levels, for people with poor sleep quality experienced decreased cognitive reevaluation or cognitive reframing of an emotional event, which helps dampen its impact^6^.

In addition, poor sleep quality may increase the risk of developing negative emotions after experiencing disruptive life events, and decrease the positive effects derived from positive life events^5^. In this sense, insomnia has been associated with suicidal ideations, suicide attempts and deaths by suicide, but it is a potentially modifiable risk factor. In fact, taking into account the current COVID-19 pandemic and the prevention measures implemented for controlling the spread of the virus, it seems that people are more prone to have suicidal ideations in conditions of social isolation and forced confinement, which is added to the fact that insomnia is considered to be one of the major predictors of suicide attempts, and has been even considered to be similar to having planned a way to commit suicide^7,8,9^.

Being exposed to the unprecedented stress situation caused by the current pandemic may make most individuals more likely to experience anxiety, depression, and disrupted sleep patterns^1^. Studies on social isolation and its effects on both, psychological well-being and sleep quality, have pointed out the occurrence of several problems, including decreased exposure to sunlight, dietary changes, room temperature changes due to confinement, reduced social interaction, overwork under stressful conditions, and living with a constant uncertainty and insecurity regarding the individual’s own health status^1,10^. Taking the above into account, the aim of this paper is to describe the possible effects on sleep quality of social isolation as measure implemented for slowing down the spread of the virus in the current COVID-19 pandemic.

## BACKGROUND

### Effects of social isolation on sleep patterns and psychological well-being.

Social isolation and confinement at home have negative effects in people, since most of them experience major changes in their daily routines due to the disruption of the activities they usually carry out outside homes, such as work and school; conversely, this situation also affects those who will have to work or study at home during the confinement period, as their daily routines at home will be also altered. Although it is possible to keep doing such activities, their performance is really distant from being optimal, since productivity and efficiency are negatively affected by the increased interaction between work and/or study life and home life. These changes may have a negative impact on the amount of time spent sleeping and staying in bed, and affect the positive associations between home, relaxation time, leisure activities, and sleep, resulting in higher levels of stress^1^.

Confinement related stress is also caused by the inability to take part in rewarding activities such as visiting family and friends, shopping, going out to restaurants, and attending cultural and sporting events^1^. Likewise, due to this confinement, some people will have less exposure to natural light, particularly those living in homes lacking good natural light (i.e., places with small windows) or without an outdoor area. In addition, many people will have fewer opportunities to exercise due to the cancellation of regular sports activities and restrictions regarding leaving home. In this context, most people will change their eating habits, particularly due to increased appetite, which is something to be expected, as this is a natural reaction to stress^11^.

Some particular situations may cause significant stress, for example, sharing a limited space in a home where pre-existing family difficulties are present may accelerate the occurrence of crises, or being elderly or living alone, since in this case, confinement is likely to aggravate the loneliness and abandonment feelings of these people^1^. Regarding sleep disorders, it should be noted that insomnia is more frequent in women^12^, and that these disorders are most commonly reported by expectant mothers or those in their postpartum period^13^. It should also be a priority considering that the social isolation period is highly challenging for children and adolescents, and it may negatively impact their sleep quality, thus, affecting their capacity to properly regulate both their behavior and emotions^1^.

Also, regarding the social isolation scenario due to the COVID-19 prevention and control measures, and their resulting feelings of loneliness, it should be noted that sleep disturbances can be both a cause and a consequence of decreased well-being and lead to work productivity related problems in the short and the medium-term, and affect the safety and health of workers in the long-term. The effects of work stress are more serious in people with little social support or who are isolated, besides they generally do not have enough resources to cope with them and to help them recover when exposed to stressful life events. Furthermore, social and emotional loneliness has been significantly associated with difficulty initiating or maintaining sleep (DIMS); in fact, recent studies have shown that while the relationship between DIMS and social isolation was entirely attributable to psychosocial stress, emotional loneliness was directly associated with DIMS if it was no related to stress^14^.

### Studies conducted on the effects of confinement during infectious diseases outbreaks prior to the COVID-19 outbreak.

Most studies on the psychological effects of confinement during infectious diseases outbreaks have not used specific instruments to assess sleep quality; also, most of them have focused on health care workers and those who were exposed to the infection or acquired it^15^. It is widely known that roughly 1 out of 6 physicians have stress-related symptoms during and after an outbreak^16^. In 2003, the psychological impact of the severe acute respiratory syndrome coronavirus (SARS-CoV) seemed to be associated with factors such as occupational role during the outbreak, training or readiness for treating patients with the disease, increased risk of contagion in the workplace, perceived risk of contagion, social support, social rejection, and the effects of the outbreak on personal or professional life^17^. Other factors associated with the psychological impact of the new coronavirus outbreak include the length of quarantines, inadequate basic supplies (food, water, electricity, etc.), shortage of personal protection equipment and reduced income^15^. In addition, in a study conducted in health care personnel (n=549), it was reported that in those having an indirect exposure to the virus, for example having friends or family members with SARS-CoV, the probabilities of having post-traumatic stress symptoms were 2-3 times higher than in those who had not had such exposure^18^.

On the other hand, taking into account that stress is one of the main causes of insomnia^19^, it is not surprising that during the SARS-CoV outbreak the prevalence of insomnia in health care workers was significantly higher than in the general population. For example, in Taiwan it was reported that 37% health workers experienced insomnia during the pandemic, while only a 4.7% prevalence rate was described for the general population^20,21^. Furthermore, sleep quality among medical personnel worsened during the outbreak, but it gradually improved after 2 weeks, suggesting that insomnia was related to the stress caused by the risk of contagion^3^.

In addition, people who experience sleep pattern disruptions related to stress are more likely to develop chronic insomnia^2^. Also, pre-existing insomnia is a relevant risk factor for the development of post-traumatic stress disorder (PTSD) when exposure to a major stressor occurs^22^, and later sleep quality is worsened since PTSD has been associated with increased sleep disruption^23^.

## COVID-19 AND INSOMNIA

Although few studies on the mental health effects in the general population of the current COVID-19 pandemic have been conducted to date, it has been reported that it has had a large negative impact, leading to a significant increase in the amount of people who experience stress related symptoms, or develop anxiety, depression, insomnia, irritability and fear^24^. In this regard, a study conducted in a large sample (n=1210) found that more than 50% of the participants reported having experienced a moderate or severe psychological impact during the COVID-10 outbreak^25^. In fact, the term coronasomnia has been proposed to describe alterations in the quality and quantity of sleep due to the COVID-19 pandemic.

In order to study sleep disorders, it is necessary to start from the conceptualization of sleep quality, which involves both quantitative aspects such as duration, latency or the number of nocturnal awakenings, and subjective qualitative aspects such as sleep depth and how much restful sleep is. It should be noted that the components of sleep quality and their individual importance vary from one person to another^26^. For this reason, in order to study this concept there are measuring instruments such as the insomnia severity index (ISI)^27^, a self-reporting questionnaire consisting of 7 items that can be answered using a 0-4 score (score range: 0-28). In this index, the cut-off point for determining the presence of insomnia is an ISI>8^28^; furthermore, according to the total score obtained, the following diagnoses can be reached: 0-7: absence of clinically significant insomnia; 8-14: subclinical insomnia; 15-21: moderate clinical insomnia; and 22-28: severe clinical insomnia^3,29,30^.

Another instrument commonly used for this purpose is the Pittsburgh sleep quality index (PSQI), a self-report questionnaire with 19 questions that measures sleep quality and sleep disturbances in the last month prior to its administration^31^. The PSQI is a short, simple and well accepted questionnaire that helps identify ‘good’ and ‘bad’ sleepers, and despite it does not provide a diagnosis *per se*, it is useful for screening possible insomnia cases^26^. Its 19 questions are grouped into seven sleep components: subjective sleep quality, sleep duration, sleep latency, usual sleep efficiency, use of medications, sleep disturbances and daytime dysfunction, and in each component a score between 0 and 3 can be obtained, thus the overall PSQI score ranges from 0-21, where a score>5 suggests poor sleep quality, and higher scores, more severe sleep disturbances^32^. Both instruments are useful tools for evaluating sleep disorders associated with the current pandemic.

### Studies on sleep disorders during the COVID-19 outbreak.

In China, a survey was conducted in 3,637 individuals between February 5 and 19, 2020 to retrospectively evaluate sleep disorders, mainly insomnia, before the outbreak by using the ISI; also, in the same study, these disorders were assessed during the outbreak (i.e., after January 21, 2020, when the first person-to-person transmission of the virus was confirmed and confinement measures were started)^33^. According to this study, during the COVID-19 outbreak, insomnia prevalence increased significantly (ISI>7, 26.2% vs. 33.7%, *p*<.001), 13.6% of the participants developed de novo insomnia and 12.5% experienced worsened insomnia related symptoms according to their ISI score. In addition, during the outbreak, the average time spent in bed increased (485.5 minutes before the outbreak vs. 531.5 minutes during the outbreak), as well as total sleep time (432.8 minutes vs. 466.9 minutes). Furthermore, during the outbreak, average bedtime and wake-up time were delayed (25.6 minutes and 71.7 minutes, respectively)^33^.

Also, another study conducted in early 2020 on 1,563 Chinese health workers, including some working in the front line against the COVID-19, reported that 36.1% (n=564/1563) had insomnia symptoms (ISI≥8)^3^. In said study, the main risk factors for insomnia were the educational level, working in an isolation unit, being concerned about getting infected with the virus, perceiving no help at all in terms of psychological support, and having a very strong uncertainty regarding the effective control of the outbreak.

Likewise, in another study conducted in Chinese health workers, it was found that almost 1 out of each 4 participants had sleep problems during the outbreak^32^, a significantly higher figure than the one observed in other occupational groups. In this regard, the heavy workloads and work intensity to which health care workers are exposed during a severe epidemic may explain these findings, as it happened before with the SARS-CoV and the Middle East Respiratory Syndrome (MERS-CoV) epidemics, since these workers have less time to rest properly and are more prone to develop chronic stress and psychological distress. In severe cases, even PTSD symptoms may occur in this population, which is highly correlated with sleep deprivation^32^.

Also, in a study on PTSD symptoms and sleep quality in medical residents (n=285) in Wuhan, China (as assessed using the PTSD checklist for DSM-5 (PCL-5) and the PSQI, respectively), it was found that those with better sleep quality or less frequent early morning awakenings reported fewer PTSD symptoms during the COVID-10 outbreak^31^; also, there was a significant association between having higher PCL-5 scores and having worse subjective sleep quality, experiencing less sleep time and having more problems to fall sleep. On the other hand, according to this study, one month after the COVID-19 outbreak started, the prevalence of PTSD, in Wuhan, one of the most affected areas in China, was 7%^31^.

In a cross-sectional study conducted in Chinese health workers (n=2182) eight weeks after the COVID-19 outbreak started in order to determine the presence of insomnia using the ISI, it was found that insomnia was more prevalent in physicians and nurses compared to non-medical workers (38.4% vs. 30.5%)^30^. In this regard, a study conducted in a similar time frame reports that poor sleep quality prevalence in front line physicians was approximately 18.4%^34^. Finally, Lai et al. (2020)^35^, in a cross-sectional study conducted in 1,257 Chinese health workers (physicians and nurses) from 34 hospitals, describe that a significant proportion of participants reported insomnia, (n=427), that is, 1 out of 3 (34%), which is a similar figure to those reported for the 2003 SARS-CoV outbreak^20,21^. Also, according to these authors, nurses (women), frontline health care workers, and those working in Wuhan, China, had more severe scores in all measurements of mental health symptoms than other health care workers; in addition, severe insomnia (ISI>22) was almost four times higher in health workers in the first line against COVID-19 compared to those in the second line (1.7% vs. 0.4%)^35^.

Findings reported above correlate with studies conducted outside of China, for example the one carried out by Ferini-Strambi et al. (2020)^36^ at the Sleep Disorders Center of the San Raffaele Scientific Institute, in Milan, Italy. In said study, out of 40 medical workers who provided health care to COVID-19 patients, 35% suffered from sleep disturbances with a sleep efficiency (SE) below 90% and PSQI scores ranging from 7 to 21. Also, a significant negative correlation between SE and PSQI (r=−0.54; *p*=0.04) was reported. Similarly, a negative association between sleep efficiency and age was found^36^. In addition, Marelli et al. (2020)^37^, in a study carried out in Milan, in 400 students and administrative staff without COVID-19 symptoms from the University Vita-Salute San Raffaele, reported an increase in the hour they went to bed to sleep (BT hr), in sleep latency (SL) and in the time they woke up in the morning (WU hr) during the pandemic. In addition, a worsening of sleep quality (according to the PSQI) and insomnia symptoms (according to the ISI) were also observed^37^. In particular, during the confinement period, students were more affected by increased SL and WU hr. In the case of administrative workers, the prevalence of sleep-maintenance insomnia before the COVID-19 pandemic began was of 24%, yet it significantly increased during the pandemic, reaching 40%. Also, before the pandemic, 15% of workers had trouble falling asleep, but during the pandemic 42% of them experienced these problems^37^.

Furthermore, Cellini et al. (2020)^38^, in a study conducted in Italy from March 24 to March 28, 2020, 1,310 individuals were asked, by means of an online survey, about the use of digital media before going to bed to sleep, their sleep patterns and their subjective experience regarding the time they spent using digital devices 2 during the week from March 17 to March 23 (the second week of the confinement period in the country) and during the first week of February (February 3-10), that is, before any restriction had been implemented in Italy. These authors found that participants increased the time they spent using digital media 2 hours before going to bed during the confinement period. Besides, their sleep time changed notably, since they reported going to bed to sleep and waking up later than usual, thus spending more time in their beds; also, they reported having a poorer quality of sleep^38^. In addition, the prevalence of poor sleep (i.e., PSQI>5) increased from 40.5% to 52.4% during lockdown, and on average, bedtime was delayed 41 minutes in both, workers and students.

The confinement restrictions had an even stronger effect on waking up times, particularly in workers, who started waking up about 1 hour and 13 minutes later than usual, while in students this delay was around 45 minutes. In general, delayed wake-up times resulted in more time spent in bed (TIB), while the restrictions were active, especially in workers, who spent 26 minutes more in bed compared to the time spent there before restrictions were imposed, in contrast to students, who in average spent only 5 more minutes. Sleep quality (global PSQI score) was more affected in participants with a high DASS-21 score, that is, those with higher symptoms of depression, anxiety, and stress^38^.

As mentioned above, sleep is an important moderating factor in the relationship between occupational stress and anxiety: stress causes sleep quality problems, and insomnia increases the perception of stress^4,39^. In particular, during the COVID-19 pandemic, health workers are at higher risk of having stress and poor sleep quality since they are subjected to a high workload and have a higher risk of being infected with the SARS-CoV-2.

In this sense, a study conducted in health workers, with and without COVID-19, and who had or had not been directly exposed to the virus, reported the risk of developing anxiety and/or depression was 4 times higher in health workers with the disease than in those who did not have it^39^. Likewise, in health workers who had been directly exposed to the virus, the risk of anxiety and depression was two times higher, being those with poor sleep quality the most affected population in both groups^39^. According to these authors, exposure to patients with COVID-19, occupational stress, and the perception of being poorly protected against contagion were factors significantly associated with the development of anxiety and depression. Another relevant finding was that the prevalence of anxiety and depression disorders reported in the study was not higher than the prevalence usually described for health workers at the same hospital in years before the COVID-19 outbreak occurred.

So it is concluded that testing positive for COVID-19 and being directly exposed to COVID-19 patients without proper personal protective equipment are major contributors to altered mental health and to developing high stress levels, occupational anxiety and depression, suggesting the SARS-CoV-2 infection risk has a direct impact on the emotions of these workers^39^.

### Altered sleep patterns in patients with mental disorders during the COVID-19 pandemic.

Hao et al. (2020)^29^, in a case-control study conducted in 96 patients with mental disorders (mainly anxiety and depression) and 109 controls (individuals without these conditions), describe that during the peak of the COVID-19 outbreak, when strict social distancing measures were implemented, the cases had significantly higher scores in the event-impact scale-revised (EIE-R), the abbreviated scales of depression, anxiety and stress (DASS-21) and the ISI instruments, and that more than 25% of them reported experiencing PTSD-like symptoms and moderate to severe insomnia. In fact, according to this study, in 27.6% (n=20) of the cases the ISI score was ≥15 (moderate to severe clinical insomnia), which was significantly higher than the 0.9% reported for the controls (n=1). In addition, the mean ISI score for the cases was 10.1, which was significantly higher than that observed for the controls (4.63)^29^.

### Studies conducted after preventive isolation ended and people started returning to work.

Tan et al. (2020)^40^, in a study conducted in n=673 Chinese workers, found that after returning to work, about 10.8% of the respondents had symptoms suggesting PTSD and that anxiety, depression, stress, and insomnia had a prevalence of 3.8%, 3.7%, 1.5%, and 2.3%, respectively^40^. Another relevant finding by this study was that in those workers who considered returning to work as a health hazard, physical symptoms, including fever, chills, headache, myalgia, cough, difficulty in breathing, dizziness, sore throat, nausea, vomiting or diarrhea, were reported and the average ISI score was higher than in those who did not deem this situation as a health hazard^40^.

## RECOMMENDATIONS TO KEEP A HEALTHY SLEEP/WAKE CYCLE

Cognitive behavioral therapy for insomnia (CBT-I) indications, which have been traditionally aimed at treating long-term or chronic insomnia problems, are appropriate during the current pandemic. Recent studies have shown that acute insomnia can be effectively treated by modifying stressful situations using a wide range of therapeutic options such as sleep hygiene education, relaxation therapy, stimulus control, sleep restriction therapy, and cognitive reevaluation^1^.

Sleep hygiene recommendations during mandatory or preventive confinement include ensuring at least 30 minutes of sunlight exposure per day to improve melatonin production, avoiding drinking coffee or tea with caffeine content at night, reducing alcohol consumption, avoiding physical activity immediately before going to bed, and ensuring optimal room temperature for getting to sleep. Regarding stimulus control, its main objective is to replace negative associations people have with their beds and bedrooms by positive associations, for example stopping doing any other activity than sleeping or having intercourse in the bed, or going to bed and getting up at the same time every day, regardless of total sleep time. Other options to treat insomnia include relaxation therapies, which focus on reducing stress levels, and may include muscle relaxation and meditation techniques.

On the other hand, cognitive reevaluation focuses on the confrontation of dysfunctional ideas or beliefs about sleep problems and, in many cases, about their causes. In this sense, cognitive control and “concern time” are addressed by allocating and spending a certain amount of time per day, and in a specific place chosen for this purpose, to work on solving concerns, make plans, and delve into daily life problems in order to prevent these thoughts from interfering with falling sleep at night. In addition, using a paradoxical intention, patients are asked to try to stay awake rather than striving to fall asleep, which in turn allows falling asleep faster since sleep drive is promoted by doing this. Finally, sleep restriction seeks to increase sleep pressure and sleep quality by gradually limiting the total time spent in bed according to the average night sleep duration, since the more time spent in bed, the more time spent failing to fall sleep^1,41^ (Table 1).

**Table 1.** Cognitive and behavioral recommendations for coping with insomnia during the COVID-19 pandemic^1^.

• If possible, get exposed to daylight, particularly in the morning, for at least 30 minutes. If this is not possible, make sure your home is well lit during daytime by opening curtains and blinds.
• Get regular physical activity, preferably in daylight.
• Choose engaging in relaxing activities before bedtime: e.g., reading a book, practicing yoga, etc.
• Do not eat within 2 hours before going to sleep.
• Avoid drinking coffee and coffee products in the evening.
• Keep a regular schedule for waking up and going to bed.
• Spend some time during the day (e.g., 15 min) to reflect on the current pandemic: write down your thoughts, talk about the stress you feel, etc.
• Use your bed only for sleeping and having sex.
• Go to bed only when you feel sleepy.
• Use social networks to share feelings of anxiety and stress with family and friends; also share positive and distracting information, e.g., humorous content, if possible COVID-19 unrelated.

Source: Author’s elaboration.

In case of severe acute insomnia due to external stressors such as confinement and quarantine measures, short-term pharmacological treatment might be effective and could be prescribed. In this regard, the current version of the European guidelines for the diagnosis and treatment of insomnia recommends using benzodiazepines and Z-hypnotics as second-line medications for treating this sleep disorder if CBT-I measures fail, as long as these are short-term treatments. Alternatively, when benzodiazepine use is not possible mainly because of potential adverse effects, or in cases of mental disorder comorbidity (e.g., anxiety or major depressive disorder), antidepressants with a sedative effect may be used^12^. In the case of health care workers dealing with the COVID-19 pandemic, several recommendations for maintaining the physiological sleep/wake cycle are presented in Table 2.

**Table 2.** Cognitive and behavioral recommendations for health workers to cope with insomnia during the COVID-19 pandemic^1^.

• Plan spending brief moments during the day with trusted colleagues or family members to express your emotions and concerns about work.
• In your free time, do activities that get you distracted from the pandemic and do not to give up on doing pleasant activities.
• Limit as much as possible the time you spend getting updates and news about the COVID-19 pandemic that are not directly related to work.
• In your free time, try to exercise regularly in the evening.
• Eat light meals, at set times if possible, and try to do it several hours before the time you want to fall sleep.
• Avoid the consumption of energy drinks.
• Avoid drinking of coffee and its derivatives.
• Avoid using psychoactive substances.
• If you experience symptoms associated with sleep deprivation or fatigue, including work-related mistakes, inability to concentrate and make decisions, extreme irritability or strong emotional reactions, please inform your colleagues and supervisors about it. Take a short break; even a short nap can help you to partially reduce these symptoms.
• After ending a long shift, in particular a night shift, do not drive home. If possible, take a taxi or a bus.
• After ending your night shift, wear dark glasses when going to your home.

Source: Author’s elaboration.

Finally, there are strategies that can be implemented to improve circadian rhythm, such as getting up at the same time every day if living in the tropics, keeping a daytime routine during the mandatory confinement period, trying to be exposed to fresh air and sunlight even through a window, getting regular exercise, avoiding naps longer than 20 minutes, and keeping doing social activities such as talking on the phone with family and friends, since these activities allow the exchange of feelings and emotions regarding confinement. Also, drinking stimulating drinks after 4 p.m. should be avoided, and after 7 p.m. only relaxing activities with low light and noise should be done; in addition, one hour before bedtime, a quiet sleep routine should be established, where any electronic screen is turned off and preparations for going to bed are made. Finally, during nighttime, a room temperature between 18-20°C should be kept, and in case of waking up at night, it is necessary to get out of bed and get back to it when feeling sleepy, since bed must only be used for sleeping and having sex^42^.

## CONCLUSION

Social isolation, as a prevention and control measure for slowing down the spread of the new coronavirus, has been associated with a higher occurrence of mental disorders in the general population, especially in health workers. Sleep alterations are among the first expected clinical manifestations caused by this situation, with a reported prevalence close to 30%, and these disorders play an important role in the development of other mental disorders. Cognitive-behavioral strategies for treating insomnia are easy to implement and are useful measures to cope with sleep pattern alterations resulting from the stress caused by the social distancing and confinement measures implemented to slow down the new coronavirus spread.
